# Selenium Concentrations in Soccer Players During a Sports Season: Sex Differences

**DOI:** 10.3390/nu17142257

**Published:** 2025-07-08

**Authors:** Víctor Toro-Román, Jesús Siquier-Coll, Francisco J. Grijota, Marcos Maynar-Mariño, Ignacio Bartolomé, María Concepción Robles-Gil

**Affiliations:** 1 Research Group in Technology Applied to High Performance and Health, Department of Health Sciences, TecnoCampus, Universitat Pompeu Fabra, 08302 Mataró, Spain; 2 Department of Communication and Education, University of Loyola Andalucía, 41704 Sevilla, Spain; 3 Faculty of Sport Sciences, University of Extremadura, 10003 Cáceres, Spain; 4 Faculty of Education, Pontifical University of Salamanca, 37007 Salamanca, Spain

**Keywords:** athletes, trace minerals, plasma, urine, erythrocytes, platelets

## Abstract

**Background**: Selenium (Se) is a trace mineral element with important roles in enhancing athletic performance and athlete recovery. **Objectives**: This study aimed to observe the differences in plasma, urinary, erythrocyte, and platelet Se concentrations between sexes and analyze the variations in Se concentrations during the soccer season. The main hypothesis was that significant differences in Se levels would be observed between male and female athletes and that these differences would fluctuate throughout the season due to varying training loads and nutritional factors. **Methods**: Twenty-two male (20 ± 2 years; 1.76 ± 0.06 m; 14.73 ± 3.13 years’ experience; fifth Spanish division) and twenty-four female soccer players (23 ± 4 years; 1.65 ± 0.06 m; 14.51 ± 4.94 years’ experience; second Spanish division) participated. Three assessments were conducted during the season. Evaluations included anthropometry, body composition, fitness (cardiorespiratory and vertical jump), and nutritional intake. Venous samples of blood and urine were obtained. The concentrations of Se in the plasma, urine, erythrocytes, and platelets were analyzed through inductively coupled plasma mass spectrometry. **Results**: No differences in Se intake were observed. The Se concentrations in the plasma, urine, and platelets were found to be higher in males, while females showed elevated levels in their erythrocytes (*p* < 0.05). Throughout the season, plasma and platelet Se concentrations exhibited a progressive increase (*p* < 0.05). **Conclusions**: Assessing Se status during the season is essential for evaluating nutritional supplementation to maintain performance given Se’s vital role in the immune and antioxidant systems.

## 1. Introduction

Selenium (Se) is an essential trace element that supports antioxidant activity and immune function. It is present in a wide range of foods, including seafood, lentils, beans, whole grains, organ meats, dairy products, and vegetables [1]. An adequate intake of selenium for people aged 15–65 years is 70 μg/day for men and 60 μg/day for women [1]. The total amount of Se in a human organism is approximately 3–20 mg. Understanding the distribution of this substance is crucial for its effective use: 30% resides in the liver, 30% in the muscle, 15% in the kidneys, and 10% in the plasma, with the remaining 15% redistributed to other tissues and organs. This knowledge helps optimize treatment and improve outcomes [2]. Se is a vital component of selenoproteins, which are essential for redox catalytic activity. In-depth knowledge of selenium’s storage and kinetics can help to detect possible deficits that may lead to a decrease in athletic performance due to its antioxidant activity. The primary biological functions of selenium can be encapsulated in two key aspects: (a) its function as an antioxidant and (b) its ability to modulate the immune system. These properties of selenium are especially significant for enhancing sports performance and facilitating recovery [3].

Vigorous physical exercise triggers an acute-phase immune response to the stress produced and consequently provokes a defense response against this situation, involving reactive oxygen species [4]. Exercise, similar to inflammation and infection, increases body temperature, serum cytokines (interleukin 1, interferon α), and circulating leukocytes. Short-term physical exercise can stimulate leukocyte mobilization, thereby increasing the concentration of leukocytes in the circulation [5]. In addition, exhaustive physical exercise augments oxidative stress, increasing the oxidative damage in various tissues, including the muscles, liver, heart, and lungs, in animals and humans [6]. It should also be noted that several defense mechanisms, including superoxide dismutase (SOD), catalase, and glutathione peroxidase (GPx), as well as other endogenous antioxidants, protect the cells against these toxic oxygen metabolites [7]. Se can also be considered a rate-limiting element in the GPx system [5].

Chronic exercise may activate the GPx system endogenously, serving as an adaptive mechanism that helps prevent the formation of free radicals [8]. Variations in glutathione responses may be observed among individuals, influenced by factors such as exercise protocols, experimental conditions, age, sex, and genetic predispositions [7,9,10,11]. Understanding these differences presents a valuable opportunity for further exploration and can enhance our approach to health and fitness. Although the role of Se in the post-exercise resting period has not been clarified, it is known to be part of the structure of the GPx system. SOD production can be controlled by antioxidants such as the GPx system [5]. An overall evaluation of the current knowledge on this topic inevitably suggests that there is an important relationship between Se and physical performance [12,13,14]. The impact of selenium on antioxidant activity is crucial to preventing the harmful effects of free radicals during physical exercise [15]. Equally, Se is associated with muscle fatigue [16]. Se is essential for preventing muscle fatigue due to its antioxidant function, especially in glutathione peroxidase (GPx) activity, which mitigates exercise-induced muscle damage. However, both selenium deficiency and excess selenium can increase muscle fatigue due to an imbalance in oxidative stress [10]. Indeed, the abundance of Se in the muscles could be crucial in understanding its correlation with muscle exhaustion during exercise. In addition, the role of selenium in the immune system underscores its significance for the overall health and nutrition of athletes [5].

Previous studies have indicated that physical exercise across various modalities leads to alterations in different biological matrices [13,17]. Research indicates that this element can undergo significant transformations at the extracellular level, affecting both urine and sweat composition [18]. Concerning its intracellular levels, Se homeostasis has been shown to be different depending on the level of training and the ambient temperature [17,19,20]. Hence, studying Se kinetics requires a multicompartmental analysis to determine whether there is any deficit related to time and nutrition [19], and therefore, at the cellular level, it is convenient to count on cells such as erythrocytes that have a half-life of 120 days [21] and platelets that have a half-life of 8–14 days [22].

Soccer is an intermittent sport that imposes extreme physical and metabolic demands on athletes [23]. Intense physical exertion, exposure to various environmental factors, and competitive pressure can lead to a state of oxidative stress in the organism, which is characterized by an imbalance between the production of reactive oxygen species and the endogenous antioxidant capacity to neutralize them [24]. A recent study on soccer players revealed that despite a similar intake compared to that in the control group, the Se levels in the erythrocytes and platelets were lower in the soccer players [19]. Nevertheless, the differences between sexes in terms of the kinetics of this metal are unknown, especially regarding women athletes, for whom the scientific literature on the effect of exercise on their Se levels is scarce. It should be noted that, as mentioned above, the selenium levels in athletes may vary by sex, similar to other minerals [25].

Thus, it is necessary to know the differences between sexes. Furthermore, the above study focused on a specific time of the season since, as mentioned above, chronic exercise can affect Se levels [19]. Hence, the aim of this article was to observe the differences in the Se concentrations between sexes and at different moments in a soccer season at the intracellular and extracellular levels.

## 2. Materials and Methods

### 2.1. The Study Design

This observational study followed a longitudinal, quasi-experimental design. Three evaluations were conducted over the course of a regular season involving two senior football teams—one male and one female—based in the city of Cáceres. Longitudinal comparisons were made between individual players and between the two teams to explore potential sex-related differences. The evaluations took place during three key periods: the first week of training (preseason; August 2021), the end of the first half of the season (midseason; January 2022), and the final week of training (end of season; May 2023).

All assessments were carried out weekly in the morning and in the same sequence for all participants, in order to minimize the influence of circadian rhythms. The evaluations were also conducted under comparable environmental conditions (a temperature between 18 and 25 °C and a relative humidity between 45% and 55%). In the two days prior to each evaluation, the participants completed a questionnaire assessing their intake of macronutrients and micronutrients. Additionally, the training load for both teams was reduced during these two days to ensure that players were able to complete the assessments with minimal fatigue.

### 2.2. The Participants

The present study included a total of forty-six football players. Prior to their participation, all individuals were informed about the aims of this study and provided their written informed consent. The study protocol was reviewed and approved by the Biomedical Ethics Committee of the University of Extremadura (Cáceres, Spain), under reference number 135/2020. This approval complied with the principles outlined in the Declaration of Helsinki, as revised at the World Medical Assembly held in Fortaleza in 2013, regarding research involving human participants. To maintain confidentiality, each participant was assigned a unique identification code for sample collection and data handling.

Participants were grouped based on their sex and the team to which they belonged (see Table 1). The male footballers played for a team competing in the fifth tier of Spanish football, while the female footballers were part of a team participating in the national second division. The details of the physical training routines for both teams are presented in Table 1. A post hoc statistical power analysis was conducted. For the sample of 46 participants, with an alpha level of 0.05 and a given effect size f, the statistical power was calculated to be 0.643.

The inclusion criteria for participation in the present study were as follows: (i) the participants must have resided in the same city for at least one month prior to and during the study; (ii) they could not have any existing medical conditions; (iii) they must not have taken any medication or supplements containing mineral elements during the study period or in the month leading up to the initial evaluation; (iv) they could not be smokers or users of recreational drugs; (v) they had to possess over five years of experience competing in soccer; (vi) they were not allowed to alter their nutritional or physical activity habits during this study; and (vii) they could not have missed training with the team for more than 30 days. Additionally, the female participants were required to meet the following criteria: (viii) they must have had regular menstrual cycles for at least six months before the start of the study and throughout its duration; (ix) they could not have experienced any issues related to their menstrual cycles; and (x) they could not be users of contraceptives.

To gather information on the characteristics of their menstrual cycles, the women’s soccer players participated in an online questionnaire. A researcher was available to assist the participants as they completed it. The questionnaire included inquiries about cycle length, duration of bleeding, bleeding patterns, age of onset, regularity of menstruation, and associated pain or symptoms. All of the women had regular menstrual cycles (Table 2)

### 2.3. Anthropometry, Body Composition, and Physical Fitness Assessments

The participants’ anthropometric features were assessed in the morning under the same conditions (fasting for 8 h from food and/or caloric beverages, while water intake was permitted) and with minimal clothing. All measurements were performed by the same evaluator and on the right side of the body following the guidelines of the Spanish Group of Cineanthropometry [26]. The evaluations carried out included stretch height, body weight, and skinfold measurements (abdominal, suprascapular, subscapular, tricipital, thigh, and calf); bone diameters (bicipital, humerus, and femur); and muscle perimeters (relaxed arm and calf). The materials utilized in this study included a wall-mounted stadiometer (Seca 220, Hamburg, Germany), an electronic digital scale (Seca 769, Hamburg, Germany), a Holtain© 610ND skinfold caliper (Holtain, Crymych, UK), a Holtain© 604 pachymeter (Holtain, Crymych, UK), and a Seca© 201 tape measure (Seca, Hamburg, Germany). Each parameter was evaluated three times, and the mean value was used for the statistical analysis. The fat percentage was calculated using the Yuhasz equation [27].

The explosive power of the lower limb was assessed through the vertical jump test. A photoelectric cell platform (Optojump, Mycrogate, Mahopac, NY, USA) facilitated the testing. The countermovement jump test (CMJ) was conducted in accordance with the protocols established by Komi and Bosco [24]. Preceding the execution of the CMJ, a warm-up was performed mobilizing the knee and hip. From this point onward, the members performed 4–5 half squats without a load and afterward an isometric squat for 5 s. Every vertical jump test was repeated twice, and the best result was used for the analysis. There was a 30 s rest between repeats. There were consistently two evaluators present to ensure that the vertical jump was performed correctly. To perform the CMJ, the participants began from an upright position, with their feet a shoulder width apart and their hands resting on their hips. Starting from this stance, the participants carried out knee flexion–extension in one continuous motion, concluding with a jump at the highest intensity achievable.

A maximal exercise stress test was conducted on a treadmill (Ergofit Trac Alpin 4000, Pirmasens, Germany) equipped with a gas analyzer (Geratherm Respiratory GMBH, Ergostik, Ref 40.400, Bad Kissingen, Germany) and a heart rate monitor (Polar^®^ H10, Kempele, Finland) to evaluate their cardiorespiratory fitness. The test protocol involved increasing running speed by 1 km/h every minute until exhaustion, starting at 7 km/h with a constant gradient of 1%. Prior to the test, the participants completed a 15 min warm-up at 6 km/h. The test was considered valid if the following criteria were met: (a) a respiratory exchange ratio (RER) above 1.05 and (b) a plateau in oxygen consumption.

### 2.4. The Nutritional Assessment

All participants completed a nutritional survey to ensure that they followed a similar diet. The survey consisted of a 3-day daily nutritional log of 2 pre-assigned weekdays and 1 weekend day. The participants reported the type, frequency, and amount (in grams) of food they consumed daily, and diet composition was assessed using food tables [28]. The investigators performed ratio conversion to estimate the intake of each nutrient using the above food tables. For accuracy, an accompanying document was sent to the participants to facilitate the estimation of the amounts of each food ingested according to the packaging used in order to reduce measurement bias. Data were obtained for macronutrients (carbohydrates, proteins, and lipids) and Se.

### 2.5. Sample Collection

Prior to the assessment days, the players were provided with a kit (container + tube) in order to collect urine during their first urination in the morning. The participants went to the laboratory on the assessment days between 8:00 and 8:30 a.m. on an empty stomach with these canisters and tubes filled.

A total of 9 mL was collected from the first urine of the day into BD Vacutainer tubes. The tubes and canisters (100 mL) of leftover urine were frozen at −80 °C until the analysis. Before the day of the analysis, all samples were thawed and homogenized through shaking.

Regarding blood collection, 15 mL of venous blood was obtained from each participant using a 20 mL plastic syringe (Injekt, Braun, Melgunsen, Germany) and a G21 sterile moth needle (Mirage Pic Solution, Trieste, Italy). Of the total, 2 mL was collected into vacutainer tubes with ethylenediaminetetraacetic acid to determine the hematological parameters, and 5 mL was collected into tubes for the biochemical and hormonal analyses. The remaining 8 mL was collected into two 4 mL vacutainer tubes with sodium citrate to determine the Se levels in the different biological compartments. Two tubes were collected for Se determination as leftover samples in case of any loss/error of analysis. The techniques for obtaining the plasma, erythrocytes, and platelets were similar to those reported in other studies previously published by the research group [19,29].

### 2.6. Hormone Sample Collection and Determination of the Hematological Parameters

Two milliliters of blood was used to determine the hematological and hormonal parameters (Progesterone and Estradiol-17β). All determinations were performed in a duly accredited, external clinical analysis laboratory in the city of Cáceres. The equipment and techniques were provided by the personnel at this laboratory.

An automatic cell counter (Coulter Electronics LTD, Model CPA; Northwell Drive, Luton, UK) was used to determine the hematological parameters. The coefficients of variation were approximately 2.0% and <1.5% for the erythrocytes and platelets, respectively. Female hormones were also analyzed in the serum using the enzyme-linked immunosorbent assay (ELISA) technique with a spectrophotometer. The sensitivity for their levels was 0.27 nmol/L, with a 0.87–205 nmol/L range.

### 2.7. Determination of Se

The techniques used to measure the Se levels in the different biological compartments were similar to those reported in other studies previously published by the research group [19,29]. The method was fully developed at the Elemental and Molecular Analysis Service within the Research Support Services of the University of Extremadura, employing inductively coupled plasma mass spectrometry (ICP-MS) (7900, Agilent Technologies, Santa Clara, CA, USA).

Calibration curves demonstrated linearity above 0.985 for the plasma, serum, urine, erythrocytes, and platelets. The instrument was calibrated using several standards prepared from commercially available certified multi-element solutions.

For the plasma, serum, and urine samples, the reagents used included 69% nitric acid (Trace Select, Fluka, Sigma-Aldrich, St. Louis, MO, USA) and ultrapure water produced using a Milli-Q system (Millipore, Burlington, MA, USA). A 400 μg/L rhodium solution was employed as the internal standard.

For the erythrocyte and platelet samples, 69% nitric acid and hydrogen peroxide (Trace Select, Fluka, Sigma-Aldrich, St. Louis, MO, USA), along with ultrapure Milli-Q water (Burlington, MA, USA), were used. A combined internal standard of 400 μg/L of yttrium and rhodium was added to these samples.

### 2.8. The Statistical Analysis

The data analysis was carried out using IBM SPSS Statistics software (version 25.0, IBM Corp., Armonk, NY, USA), with the results expressed as the mean ± standard deviation. A post hoc power analysis was performed using the GPower software (version 3.1). The Shapiro–Wilk test was employed to assess the normality of the variable distributions, while the Levene test was used to evaluate the homogeneity of variances.

The two-way ANOVA test (the sex effect and the assessment effect) was used to show the differences when both groups were evaluated (the sex effect) in the different assessments (the assessment effect), as well as the interaction between the two independent variables (assessment and sex). A one-way ANOVA test was utilized to analyze the variations in the assessments of female hormones. To assess the variables (time points in the longitudinal analysis), the Bonferroni post hoc test was applied to determine the specific differences since more than two assessments were performed.

The effect size for the Se concentrations was evaluated using partial eta squared (η^2^), where 0.01–0.06 indicates a small effect, 0.06–0.14 a moderate effect, and >0.14 a large effect [30]. Differences with *p*-values of ≤0.05 and ≤0.01 were regarded as statistically significant and highly significant, respectively.

## 3. Results

The results obtained in the present study are shown below. Table 3 shows the characteristics of the participants. There were differences between the sexes in body weight, fat percentage, VO_2max_, the CMJ, and erythrocytes (*p* < 0.05). Moreover, significant changes were observed throughout the season in fat percentage, VO_2max_, and erythrocytes (*p* < 0.05).

Table 4 shows the nutritional intake of macronutrients and Se throughout the season. There were differences between the sexes in their protein intake, being higher in the men’s soccer players (*p* < 0.05).

Table 5 shows the concentrations of female hormones (progesterone and estradiol) during the sports season. No significant differences were reported.

The intracellular and extracellular Se concentrations are shown in Table 6. Significant changes between sexes were reported in all of the concentrations analyzed (*p* < 0.05), with higher Se concentrations in the plasma, urine, and platelets (*p* < 0.05) in the men. On the other hand, the women showed higher Se concentrations in their erythrocytes (*p* < 0.05). Regarding the differences throughout the season, significant differences were reported in the plasma, urine, and platelets (*p* < 0.05). Specifically, there was a progressive increase in Se in the plasma and platelets, while in the urine, there was a decrease below the initial values.

## 4. Discussion

The purpose of the present investigation was to observe the differences in the Se concentrations between sexes and at different times during a soccer season at the intracellular and extracellular levels. Higher selenium levels were observed in the plasma, urine, and platelets in men, whereas women showed higher levels in the erythrocytes. The selenium concentrations varied throughout the season, increasing in the plasma and platelets and decreasing in the urine. These findings demonstrate clear sex differences and seasonal variations in selenium status among soccer players.

Regarding the performance outcomes, explosive vertical jump strength is crucial to soccer performance [31]. The CMJ is related to performance in different tests in elite soccer teams [32,33]. In the present study, there were no significant changes in the CMJ. These results are contradictory to those reported in Israeli [33] and English [34] male soccer players. The performance in vertical jumping and muscle strength could be maintained or increased throughout the season due to the regular execution of strength- and plyometric-oriented training [35]. The values observed in female and male soccer players were higher than those reported by Loturco et al. [36] in Brazilian male senior players. The sex differences observed in the CMJ in the current study are in agreement with previous research in Italian soccer players [37].

Well-developed aerobic fitness enables soccer players to sustain high-intensity repetitive actions during a match and to accelerate their recovery process [31]. In relation to the above, Wisloeff et al. [35] demonstrated a significant difference in VO_2max_ between the highest and lowest ranked elite teams. The results obtained in VO_2max_ in male players is within the range reported in the meta-analysis by Slimani et al. [38]. However, the female players presented lower values compared to those in other studies [39,40]. Regarding the evolution throughout the season, Kalapotharakos et al. [41] reported increases in VO_2max_ and maximal velocity achieved in Greek male soccer players. Similarly, Metaxas et al. [42] reported enhancements in VO_2max_ after the preseason. Regarding the evolution of VO_2max_ throughout the season, significant increases were observed between the first and second evaluations in both sexes, which could be attributed to the effect of accumulated training during the preseason. However, at the third evaluation, these values did not continue to increase, which could reflect a stabilization of aerobic performance or even slight accumulated fatigue at the end of the season. This pattern has been previously described by Kalapotharakos et al. [41], who observed improvements in VO_2max_ after the preseason, but these improvements were not necessarily maintained in subsequent phases. These findings underline the importance of appropriately periodizing the training load to sustain physiological adaptations throughout the competitive calendar.

In this research, both groups ingested levels of Se above the RDI (55 μg/day) [43]. Specifically, the mean values ranged from 136 to 159 µg/day, representing an intake approximately 2.5 times higher than the RDI. This high intake could be related to a diet rich in foods high in Se, such as fish, shellfish, whole grains, and nuts, although the specific dietary sources were not identified. It is noteworthy that previous studies in the Spanish population have reported average Se intakes close to 60–100 µg/day, suggesting that the participants in this study had a more favorable dietary profile regarding this micronutrient. In comparison to the present study, McCrink et al. reported a lower intake in Gaelic soccer players [44]. Alternatively, Anđelković et al. report a higher intake in Serbian soccer players, as did another recent study [19,45]. A study conducted in 553 elite German athletes of different modalities observed deficiencies in their Se intake [46]. Athletes require a higher Se intake to increase the functionality and activity of their antioxidant systems [47]. Furthermore, the adaptive response of the antioxidant system to physical training is also dependent on nutritional factors [48].

Regarding biomarkers, Se status can be assessed using a spectrum of methods, including measurements in the whole blood, plasma, serum, urine, red blood cells, platelets, hair, and nails [49]. Data from 18 studies on Se supplementation included in a systematic review suggest that Se in the plasma, erythrocytes, and whole blood; plasma selenoprotein P; and the GPx activity in the plasma, platelets, and whole blood are likely to be useful markers of Se status. There was insufficient evidence for other biomarkers such as urinary Se, plasma thyroxine and plasma total homocysteine, and muscle GPx activity [50]. Notably, the plasma concentrations are similar in both sexes to those reported by Lu et al. using a similar technique [51]. However, the erythrocyte values in both men and women are lower than those reported by this same article. In contrast, the levels in men are in agreement with a previous study [19]. The evaluation of Se biomarkers depends on the objective for evaluation with respect to Se metabolism [52]. The most commonly used marker for assessing Se status, particularly in humans, is plasma Se (or serum Se). Plasma or serum Se and platelets reflect short-term status, whereas the red blood cells are indicators of long-term and past-time status. Based on Thomson, plasma or serum values are considered the preferred tool for comparing the Se status among different countries [53]. Se-containing proteins or enzymes will increase during supplementation in deficient individuals but will reach a maximum level. This makes these tests poor indicators of exposure to higher levels of Se [49]. Reports from previous researchers have indicated a relationship between Se, antioxidant activity, and exercise. Ji et al. [10] studied the effect of Se deficiency on the oxidative enzymes in the liver and skeletal muscle in chronic and acute exercise and established that Se deficiency decreased the GPx concentrations in the liver and muscle [5]. Se deficiency is also known to cause muscle fatigue in exercising individuals [54].

Plasma Se, even though it is generally not considered an ideal biomarker of Se status because it is susceptible to changes due to sweating, is the most widely used in the literature [53,55]. In general, plasma Se increases after supplementation and could be useful as a biomarker in adults of both sexes. In addition, plasma Se reflects changes in intake in subjects with an intermediate or high Se level. Consequently, plasma Se is also likely to be useful for assessing Se status in depleted individuals. Alternatively, serum Se concentrations could reflect Se status with health effects [53]. However, interpretation of the results could be difficult for participants with a systemic inflammatory response. Erythrocyte Se is used as a marker of long-term Se status [56]. Previous authors reported lower plasma Se concentrations after 4 weeks of aerobic training [57], while in men’s soccer players, one study found an increase in Se at week 12 with respect to the preseason baseline [58]. Similarly, in this study, significant increases in plasma Se were found throughout the season in both men and women. Pograjc et al. also observed an increase in plasma Se after three months of training in military men [59]. These authors reported a decrease in the Se concentrations in the erythrocytes. In the current study, the gradual decrease in men and women between the first and second measurements did not become significant; however, a medium effect size was observed. This could indicate a possible flow from erythrocyte Se to plasma Se, as mentioned above. However, previous studies found no changes in the glutathione peroxidase activity in the erythrocytes after 4.5 weeks of training in marathon runners [60]. Another possible factor could be that physical exercise triggers Se transfer to the tissues [17,61]. The post-exercise inflammatory process could release Se to counteract the activity of reactive oxygen species since this process, together with muscle damage, is related to a fall in extracellular concentrations [54], in turn triggering this increase in erythrocyte flux to the plasma. Interestingly, in women, it had increased at the last measurement. No prior studies have been found in the scientific literature with which to compare these results in women. Further studies on the behavior of this mineral in women are necessary to establish comparisons. Another explanation for the increase in plasma Se concentrations could be the saturation of Se reserves. It is known that the amount of Se incorporated into the muscles, the main Se reserve, is saturated with a Se-rich diet [47]. Both sexes presented a Se-rich diet, which could have induced a saturated Se reserve, as mentioned above.

Regarding the urinary Se concentrations, previous authors reported lower urinary Se levels in athletes after a maximal incremental test and a 6-month aerobic training period [12,14]. This decrease in urinary elimination could be related to a possible adaptive mechanism to avoid further Se losses [14]. Soccer has a large aerobic component, so such a decrease in excretion may occur as an adaptation to training. However, this decrease in urinary Se also occurs acutely in men athletes [45]. This decrease in Se excretion was accompanied by a gradual increase in platelet Se excretion in both sexes, which also could be an adaptive mechanism to the antioxidant activity of soccer. This factor, combined with a high intake of Se, may contribute to increased levels of this mineral in the platelets. However, there are currently no studies in the scientific literature to support this observation. Further research is required to assess the selenium content in both men and women throughout a seasonal cycle.

Concerning sex differences, it has been previously reported in different populations, specifically Polish [62], Korean [63], American [64], and Italian [65], that men have higher plasma Se concentrations. According to previous authors, these sex differences could be due to differences in dietary intake of these elements; differences in occupational exposure; and differences in metabolism, including absorption, distribution, and excretion rates [64]. Also, hormonal status could partly explain the sex differences observed. Verlinden et al. [66] reported that women taking oral contraceptives had higher serum Se concentrations than those in other premenopausal groups. This observation is consistent with the strong positive relationship between plasma estrogens and plasma Se concentrations [67].

In studies analyzing zinc, men had higher concentrations in their plasma and urine, while women showed higher levels in their erythrocytes and platelets. This pattern is similar to that observed in our study for selenium, suggesting that there may be a common physiological trend in the distribution of trace elements between sexes, possibly related to differences in muscle mass, metabolism, and sex hormones [68].

In studies similar to ours, higher plasma and urine concentrations of manganese were observed in men, and higher erythrocyte levels were seen in women, which is consistent with our findings for selenium. However, the study on Mn reported a progressive decrease in the plasma and an increase in the erythrocytes in men, while our study on Se showed a progressive increase in the plasma and a decrease in the erythrocytes. This difference could be explained by the fact that Mn is not found in mature erythrocytes (without mitochondria), while Se is part of the active enzymes in these cells, such as glutathione peroxidase [69].

In this regard, higher urinary excretion was observed in the men’s soccer players at assessments 2 and 3 compared to that in women’s soccer players. The differences in Se excretion between men and women add to the list of sex-specific differences in hepatic Se metabolism which have been observed in both rodents and humans [70]. These results are in agreement with those reported in a Japanese population in whom the influence of Se intake on urinary Se excretion was evaluated [71]. Similarly, other authors have reported higher concentrations of Se in the urine in men compared to those in women [72].

Lastly, it was observed that the women’s soccer players showed a higher amount of Se in their erythrocytes, both in absolute values and relative to the number of cells. These data are in line with a study which analyzed the differences in Se status based on age, sex, and race [73].

An impact of sex on circulating Se biomarkers in animals has been observed since the late 1960s [74]. In humans, it has been recognized that women have higher circulating GPx activity than that in men but not increased Se levels [75], as in mice [76]. Subsequently, researchers determined that for individuals with the same Se intake, women had a significant increase in Se-bound albumin, whereas men had a higher proportion of total Se [77].

The findings of this study highlight the importance of monitoring Se levels in athletes, particularly to identify potential deficiencies or imbalances that could impact performance and recovery. Athletic trainers and nutritionists should consider incorporating regular Se assessments into their athlete monitoring routines, especially during periods of intense training or competition. Adjustments to dietary intake or supplementation may be necessary to optimize Se status, which could contribute to improved performance, reduced fatigue, and enhanced recovery. Tailored nutritional strategies based on individual Se levels could help support athletes’ antioxidant defenses and overall health.

This study presents certain limitations that must be considered. Firstly, the sample size was relatively small and comprised only football players from specific competitive tiers, which may limit the extent to which these findings can be generalized to athletes from other sports or with different levels of performance. Second, its observational design restricts the ability to infer causal relationships between selenium concentrations and athletic performance or fatigue. Third, factors such as menstrual cycle phases in female athletes, although monitored, could still influence selenium metabolism and were not fully controlled. Additionally, this study is limited by the availability of selenium biomarkers, which may not fully reflect selenium status. Factors such as diet, environmental exposures, and training loads could have influenced selenium metabolism. Finally, this study did not include direct measures of oxidative stress or muscle fatigue markers, limiting the ability to directly link selenium status with physiological outcomes.

## 5. Conclusions

There are differences between sexes in plasma, urinary, erythrocyte, and platelet Se concentrations. Men’s soccer players presented higher concentrations in the plasma, urine, and platelets, while women’s soccer players reported higher concentrations in the erythrocytes. Furthermore, the Se concentrations can change considerably throughout the sports season in soccer players, with its concentrations progressively increasing in the plasma and platelets and decreasing at the end of the season in the urine.

Assessing Se status throughout the season is crucial to determining the potential necessity for nutritional supplementation and upholding performance, as Se is essential for the immune and antioxidant systems.

## Figures and Tables

**Table 1:** Characteristics of the training sessions and participants.

		Men Soccer Players (*n* = 22)	Women Soccer Players (*n* = 24)
**Age (years)**		20 ± 2	23 ± 4
**Height (m)**		1.76 ± 0.06	1.65 ± 0.06
**Weight (kg)**		71.50 ± 5.93	59.58 ± 7.17
**Σ6 Skinfold (mm)**		60.34 ± 12.35	94.62 ± 18.54
**Experience (years)**		14.73 ± 3.13	14.51 ± 4.94
**Position on the Field (%)**	Goalkeeper	7.70	11.10
	Defender	30.80	33.30
	Midfielder	38.50	29.60
	Forward	23.10	25.90
**Total Training (weeks)**		36	39
**Total Training (nº)**		128.27 ± 18.59	133.54 ± 25.86
**Total Training (min)**		11,814.23 ± 1673.40	10,578.46 ± 3227.80
**Absence from Training (days)**		12.07 ± 9.34	14.14 ± 10.79

**Table 2:** The characteristics of the menstrual cycles of the women’s soccer players.

	Women Soccer Players
**Age of onset (years)**	13.5 ± 1.15
**Regular menses (%)**	100.00
**Duration of bleeding (days)**	4.77 ± 1.47
**Menstrual cycle (days)**	27.93 ± 2.78
**Use of contraceptive methods (%)**	0

**Table 3:** Anthropometry, body composition, physical condition, and hematology characteristics of the study participants.

		Men Soccer Players	Women Soccer Players	Sex Effect	Measured Effect	Sex × Measured
**Weight (kg)**	Preseason	71.50 ± 5.93	59.58 ± 7.17	<0.001	0.748	0.931
	Midseason	71.95 ± 5.87	60.44 ± 6.77			
	End of season	72.80 ± 5.68	66.39 ± 8.99			
**Fat (%)**	Preseason	9.46 ± 1.30	18.16 ± 2.74	<0.001	0.005	0.007
	Midseason	9.45 ± 1.31	15.56 ± 2.16 *			
	End of season	9.14 ± 1.23	16.54 ± 2.68			
**VO_2max_ (mL/min/kg)**	Preseason	52.21 ± 2.91	39.72 ± 6.22	<0.001	0.032	0.268
	Midseason	54.79 ± 3.70 *	42.32 ± 4.19 *			
	End of season	53.30 ± 5.11	41.06 ± 4.51			
**CMJ (cm)**	Preseason	56.94 ± 6.39	40.21 ± 7.46	<0.001	0.571	0.717
	Midseason	55.34 ± 4.72	39.70 ± 4.18			
	End of season	56.05 ± 6.39	41.45 ± 5.80			
**Erythrocytes (millions)**	Preseason	4.92 ± 0.36	4.37 ± 0.22	<0.001	0.031	0.063
	Midseason	4.83 ± 0.32 **	4.19 ± 0.27 **			
	End of season	4.99 ± 0.29 ^++^	4.35 ± 0.27 ^++^			
**Platelets (thousands)**	Preseason	204.50 ± 57.65	196.00 ± 38.01	0.274	0.542	0.222
	Midseason	196.60 ± 39.79	219.08 ± 34.19			
	End of season	195.13 ± 37.82	204.39 ± 31.52			

* *p* < 0.05L: differences between the 1st and 2nd assessments; ** *p* < 0.01: differences between preseason and midseason; ^++^ *p* < 0.01 differences between preseason and end of season.

**Table 4:** Protein, lipid, carbohydrate, and Se intake during the sports season.

		Men Soccer Players	Women Soccer Players	Sex Effect	Measured Effect	Sex × Measured
**Proteins (g/day)**	Preseason	106.1 ± 25.5	90.4 ± 21.6	0.047	0.469	0.218
	Midseason	115.5 ± 23.4	96.2 ± 18.3			
	End of season	108.9 ± 24.8	92.6 ± 20.4			
**Proteins (g/kg/day)**	Preseason	1.53 ± 0.35	1.42 ± 0.31	0.061	0.571	0.317
	Midseason	1.56 ± 0.41	1.39 ± 0.17			
	End of season	1.50 ± 0.29	1.40 ± 0.31			
**Lipids (g/day)**	Preseason	54.8 ± 19.1	48.3 ± 12.3	0.116	0.241	0.471
	Midseason	64.1 ± 15.4	55.6 ± 15.3			
	End of season	58.6 ± 17.4	60.3 ± 20.6			
**Lipids (g/kg/day)**	Preseason	0.54 ± 0.14	0.51 ± 0.11	0.248	0.366	0.589
	Midseason	0.57 ± 0.25	0.58 ± 0.15			
	End of season	0.53 ± 0.17	0.62 ± 0.21			
**Carbohydrates (g/day)**	Preseason	231.0 ± 69.1	206.1 ± 81.3	0.471	0.856	0.683
	Midseason	235.8 ± 60.3	241.5 ± 56.1			
	End of season	242.0 ± 57.0	235.8 ± 61.7			
**Carbohydrates (g/kg/day)**	Preseason	4.27 ± 1.60	4.11 ± 1.34	0.372	0.215	0.481
	Midseason	4.33 ± 1.91	4.20 ± 1.80			
	End of season	4.40 ± 1.38	4.34 ± 1.59			
**Se (µg/day)**	Preseason	150.0 ± 61.1	136.6 ± 71.6	0.141	0.920	0.538
	Midseason	156.0 ± 31.7	141.7 ± 69.2			
	End of season	159.0 ± 81.7	140.6 ± 49.5			

Se: selenium.

**Table 5:** Female hormones during the sport season.

		Women Soccer Players	*p*
**Progesterone (ng/mL)**	Preseason	2.65 ± 3.88	0.998
	Midseason	2.38 ± 3.21	
	End of season	2.31 ± 2.89	
**Estradiol-17β (pg/mL)**	Preseason	74.04 ± 45.30	0.894
	Midseason	71.32 ± 39.25	
	End of season	68.30 ± 40.93	

**Table 6:** Se concentrations in plasma, urine, erythrocytes, and platelets (absolute values and values relative to cell number).

		Men Soccer Players	Women Soccer Players	Sex Effect	Measured Effect	Sex × Measured
**Plasma (µg/L)**	Preseason	80.79 ± 18.90	70.44 ± 19.66	0.001 ^##^	<0.001 ^##^	0.189
	Midseason	88.88 ± 12.19 ^^	80.86 ± 15.28 ^^			
	End of season	116.50 ± 16.20 **	97.25 ± 16.29 **			
**Urine (µg/L)**	Preseason	24.09 ± 14.13	29.97 ± 14.92	0.006 ^##^	<0.001 ^##^	0.178
	Midseason	33.90 ± 16.53 ^^	12.95 ± 10.66 ^^			
	End of season	14.30 ± 5.79 **	9.43 ± 6.35 **			
**Absolute erythrocytes** **(µg/L)**	Preseason	65.23 ± 25.57	70.96 ± 32.94	<0.001 ^##^	0.085 ^#^	0.078
	Midseason	54.58 ± 25.40	77.23 ± 52.14			
	End of season	46.15 ± 33.08	120.90 ± 32.39			
**Relative erythrocytes** **(pg/cell^−6^)**	Preseason	14.98 ± 4.95	16.41 ± 7.01	<0.001 ^##^	0.212	0.097 ^#^
	Midseason	12.48 ± 5.51 ^^	16.96 ± 12.17 ^^			
	End of season	9.58 ± 6.99 **	26.98 ± 7.09 **			
**Absolute platelets** **(µg/L)**	Preseason	5.84 ± 2.25	4.36 ± 2.47	<0.001 ^##^	<0.001 ^##^	0.312
	Midseason	9.07 ± 2.30	6.70 ± 1.66			
	End of season	11.34 ± 4.16 **	8.41 ± 3.78 **			
**Relative platelets** **(pg/cell^−3^)**	Preseason	0.033 ± 0.013	0.023 ± 0.014	<0.001 ^##^	<0.001 ^##^	0.056 ^##^
	Midseason	0.045 ± 0.012	0.032 ± 0.008			
	End of season	0.085 ± 0.019 **	0.059 ± 0.013 **			

^##^: large effect size (>0.14); ^#^: moderate effect size (0.06–0.14); ** *p* ≤ 0.01: differences between preseason and end of season; ^^ *p* ≤ 0.01: differences between midseason and end of season; Se: selenium.

## Data Availability

The original contributions presented in this study are included in the article. Further inquiries can be directed to the corresponding authors.

## References

[1] Mehdi Y., Hornick J.-L., Istasse L., Dufrasne I. (2013). Selenium in the Environment, Metabolism and Involvement in Body Functions. Molecules.

[2] Mistry H.D., Pipkin F.B., Redman C.W.G., Poston L. (2012). Selenium in Reproductive Health. Am. J. Obstet. Gynecol..

[3] Fernández-Lázaro D., Fernandez-Lazaro C.I., Mielgo-Ayuso J., Navascués L.J., Córdova Martínez A., Seco-Calvo J. (2020). The Role of Selenium Mineral Trace Element in Exercise: Antioxidant Defense System, Muscle Performance, Hormone Response, and Athletic Performance. A Systematic Review. Nutrients.

[4] Simioni C., Zauli G., Martelli A.M., Vitale M., Sacchetti G., Gonelli A., Neri L.M. (2018). Oxidative Stress: Role of Physical Exercise and Antioxidant Nutraceuticals in Adulthood and Aging. Oncotarget.

[5] Baltaci A., Mogulkoc R., Akil M., Bicer M. (2016). Selenium—Its Metabolism and Relation to Exercise. Pak. J. Pharm. Sci..

[6] Clarkson P.M., Thompson H.S. (2000). Antioxidants: What Role Do They Play in Physical Activity and Health?. Am. J. Clin. Nutr..

[7] Bouzid M.A., Filaire E., Matran R., Robin S., Fabre C. (2018). Lifelong Voluntary Exercise Modulates Age-Related Changes in Oxidative Stress. Int. J. Sports Med..

[8] Mena P., Maynar M., Gutierrez J.M., Maynar J., Timon J., Campillo J.E. (1991). Erythrocyte Free Radical Scavenger Enzymes in Bicycle Professional Racers. Adaptation to Training. Int. J. Sports Med..

[9] Akimoto A.K., Ana Luisa M.-V., Penha Cristina Zaidan A., Luiz Carlos da Silva P., Graciana Souza L., Cassia de Oliveira H., da Silva I.C.R., Cesar Koppe G., Klautau-Guimarães M.d.N. (2010). Evaluation of Gene Polymorphisms in Exercise-Induced Oxidative Stress and Damage. Free Radic. Res..

[10] Ji L.L., Stratman F.W., Lardy H.A. (1988). Antioxidant Enzyme Systems in Rat Liver and Skeletal Muscle: Influences of Selenium Deficiency, Chronic Training, and Acute Exercise. Arch. Biochem. Biophys..

[11] Powers S.K., Jackson M.J. (2008). Exercise-Induced Oxidative Stress: Cellular Mechanisms and Impact on Muscle Force Production. Physiol. Rev..

[12] Maynar M., Muñoz D., Alves J., Barrientos G., Grijota F.J., Robles M.C., Llerena F. (2018). Influence of an Acute Exercise Until Exhaustion on Serum and Urinary Concentrations of Molybdenum, Selenium, and Zinc in Athletes. Biol. Trace Elem. Res..

[13] Maynar M., Llerena F., Bartolomé I., Alves J., Robles M.-C., Grijota F.-J., Muñoz D. (2018). Seric Concentrations of Copper, Chromium, Manganesum, Nickel and Selenium in Aerobic, Anaerobic and Mixed Professional Sportsmen. J. Int. Soc. Sports Nutr..

[14] Maynar M., Bartolomé I., Alves J., Barrientos G., Grijota F.J., Robles M.C., Munõz D. (2019). Influence of a 6-Month Physical Training Program on Serum and Urinary Concentrations of Trace Metals in Middle Distance Elite Runners. J. Int. Soc. Sports Nutr..

[15] Clemente-Suárez V.J., Bustamante-Sanchez Á., Mielgo-Ayuso J., Martínez-Guardado I., Martín-Rodríguez A., Tornero-Aguilera J.F. (2023). Antioxidants and Sports Performance. Nutrients.

[16] Fodor J., Al-Gaadi D., Czirják T., Oláh T., Dienes B., Csernoch L., Szentesi P. (2020). Improved Calcium Homeostasis and Force by Selenium Treatment and Training in Aged Mouse Skeletal Muscle. Sci. Rep..

[17] Maynar M., Grijota F.J., Siquier-Coll J., Bartolome I., Robles M.C., Muñoz D. (2020). Erythrocyte Concentrations of Chromium, Copper, Manganese, Molybdenum, Selenium and Zinc in Subjects with Different Physical Training Levels. J. Int. Soc. Sports Nutr..

[18] Siquier-Coll J., Bartolomé I., Pérez-Quintero M., Muñoz D., Robles M.C., Maynar-Mariño M. (2020). Influence of a High-Temperature Programme on Serum, Urinary and Sweat Levels of Selenium and Zinc. J. Therm. Biol..

[19] Toro-Román V., Bartolomé I., Siquier-Coll J., Robles-Gil M.C., Muñoz D., Maynar-Mariño M. (2022). Analysis of Intracellular and Extracellular Selenium Concentrations: Differences According to Training Level. Nutrients.

[20] Siquier-Coll J., Bartolomé I., Perez-Quintero M., Grijota F.J., Arroyo J., Muñoz D., Maynar-Mariño M. (2019). Serum, Erythrocyte and Urinary Concentrations of Iron, Copper, Selenium and Zinc Do Not Change during an Incremental Test to Exhaustion in Either Normothermic or Hyperthermic Conditions. J. Therm. Biol..

[21] Piomelli S., Seaman C. (1993). Mechanism of Red Blood Cell Aging: Relationship of Cell Density and Cell Age. Am. J. Hematol..

[22] Neve J. (1995). Human Selenium Supplementation as Assessed by Changes in Blood Selenium Concentration and Glutathione Peroxidase Activity. J. Trace Elem. Med. Biol..

[23] Bangsbo J. (1994). The Physiology of Soccer—With Special Reference to Intense Intermittent Exercise. Acta Physiol. Scand. Suppl..

[24] Stankiewicz B., Cieślicka M., Kujawski S., Piskorska E., Kowalik T., Korycka J., Skarpańska-Stejnborn A. (2021). Effects of Antioxidant Supplementation on Oxidative Stress Balance in Young Footballers-a Randomized Double-Blind Trial. J. Int. Soc. Sports Nutr..

[25] Toro-Román V., Robles-Gil M.C., Muñoz D., Bartolomé I., Siquier-Coll J., Maynar-Mariño M. (2022). Extracellular and Intracellular Concentrations of Molybdenum and Zinc in Soccer Players: Sex Differences. Biology.

[26] Porta J., Galiano D., Tejedo A., González J.M., Esparza Ros F. (1993). Valoración de La Composición Corporal: Utopías y Realidades. Manual de Cineantropometría.

[27] Yuhasz M.S. (1974). Physical Fitness Manual.

[28] Moreiras O., Carbajal A., Cabrera L., Cuadrado C. (2016). Tablas de Composicion de Alimentos: Guia de Prácticas.

[29] Toro-Román V., Muñoz D., Maynar-Mariño M., Clemente-Gil S., Robles-Gil M.C. (2023). Sex Differences in Copper Concentrations during a Sports Season in Soccer Players. Nutrients.

[30] Hopkins W.G., Marshall S.W., Batterham A.M., Hanin J. (2009). Progressive Statistics for Studies in Sports Medicine and Exercise Science. Med. Sci. Sports Exerc..

[31] Stølen T., Chamari K., Castagna C., Wisløff U. (2005). Physiology of Soccer. Sports Med..

[32] Mujika I., Santisteban J., Impellizzeri F.M., Castagna C. (2009). Fitness Determinants of Success in Men’s and Women’s Football. J. Sports Sci..

[33] Meckel Y., Doron O., Eliakim E., Eliakim A. (2018). Seasonal Variations in Physical Fitness and Performance Indices of Elite Soccer Players. Sports.

[34] Caldwell B.P., Peters D.M. (2009). Seasonal Variation in Physiological Fitness of a Semiprofessional Soccer Team. J. Strength Cond. Res..

[35] Wisløff U., Castagna C., Helgerud J., Jones R., Hoff J. (2004). Strong Correlation of Maximal Squat Strength with Sprint Performance and Vertical Jump Height in Elite Soccer Players. Br. J. Sports Med..

[36] Loturco I., Jeffreys I., Abad C.C.C., Kobal R., Zanetti V., Pereira L.A., Nimphius S. (2020). Change-of-Direction, Speed and Jump Performance in Soccer Players: A Comparison across Different Age-Categories. J. Sports Sci..

[37] Castagna C., Castellini E. (2013). Vertical Jump Performance in Italian Male and Female National Team Soccer Players. J. Strength Cond. Res..

[38] Slimani M., Znazen H., Miarka B., Bragazzi N.L. (2019). Maximum Oxygen Uptake of Male Soccer Players According to Their Competitive Level, Playing Position and Age Group: Implication from a Network Meta-Analysis. J. Hum. Kinet..

[39] Esco M.R., Snarr R.L., Flatt A., Leatherwood M., Whittaker A. (2014). Tracking Changes in Maximal Oxygen Consumption with the Heart Rate Index in Female Collegiate Soccer Players. J. Hum. Kinet..

[40] Miller T., Thierry-Aguilera R., Congleton J.J., Amendola A.A. (2007). Seasonal Changes in VO2max among Division 1A Collegiate Women Soccer Players. J. Strength Cond. Res..

[41] Kalapotharakos V.I., Ziogas G., Tokmakidis S.P. (2011). Seasonal Aerobic Performance Variations in Elite Soccer Players. J. Strength Cond. Res..

[42] Metaxas T., Sendelides T., Koutlianos N., Mandroukas K. (2006). Seasonal Variation of Aerobic Performance in Soccer Players According to Positional Role. J. Sports Med. Phys. Fit..

[43] Calleja C.A., Hurtado M.M.C., Daschner Á., Escámez P.F., Abuín C.M.F., Pons R.M.G., Fandos M.E.G., Muñoz M.J.G., López-García E., Vinuesa J.M. (2019). Informe Del Comité Científico de La Agencia Española de Seguridad Alimentaria y Nutrición (AESAN) Sobre Ingestas Nutricionales de Referencia Para La Población Española. Rev. Com. Científico AESAN.

[44] McCrink C.M., McSorley E.M., Grant K., McNeilly A.M., Magee P.J. (2021). An Investigation of Dietary Intake, Nutrition Knowledge and Hydration Status of Gaelic Football Players. Eur. J. Nutr..

[45] Anđelković M., Baralić I., Đorđević B., Stevuljević J.K., Radivojević N., Dikić N., Škodrić S.R., Stojković M. (2015). Hematological and Biochemical Parameters in Elite Soccer Players during a Competitive Half Season. J. Med. Biochem..

[46] Wardenaar F., Brinkmans N., Ceelen I., Van Rooij B., Mensink M., Witkamp R., De Vries J. (2017). Micronutrient Intakes in 553 Dutch Elite and Sub-Elite Athletes: Prevalence of Low and High Intakes in Users and Non-Users of Nutritional Supplements. Nutrients.

[47] Margaritis I., Rousseau A., Hininger I., Palazzetti S., Arnaud J., Roussel A.-M. (2005). Increase in Selenium Requirements with Physical Activity Loads in Well-Trained Athletes Is Not Linear. BioFactors.

[48] Margaritis I., Palazzetti S., Rousseau A.S., Richard M.J., Favier A. (2003). Antioxidant Supplementation and Tapering Exercise Improve Exercise-Induced Antioxidant Response. J. Am. Coll. Nutr..

[49] Driskell J., Wolinsky I. (2005). Sports Nutrition: Vitamins and Trace Elements.

[50] Ashton K., Hooper L., Harvey L.J., Hurst R., Casgrain A., Fairweather-Tait S.J. (2009). Methods of Assessment of Selenium Status in Humans: A Systematic Review. Am. J. Clin. Nutr..

[51] Lu Y., Ahmed S., Harari F., Vahter M. (2015). Impact of Ficoll Density Gradient Centrifugation on Major and Trace Element Concentrations in Erythrocytes and Blood Plasma. J. Trace Elem. Med. Biol..

[52] Combs F. (2015). Biomarkers of Selenium Status. Nutrients.

[53] Thomson C. (2004). Assessment of Requirements for Selenium and Adequacy of Selenium Status: A Review. Eur. J. Clin. Nutr..

[54] Milias G.A., Nomikos T., Fragopoulou E., Athanasopoulos S., Antonopoulou S. (2006). Effects of Baseline Serum Levels of Se on Markers of Eccentric Exercise-Induced Muscle Injury. Biofactors.

[55] Thomson C. (2004). Selenium and Iodine Intakes and Status in New Zealand and Australia. Br. J. Nutr..

[56] Gibson R.S. (2005). Principles of Nutritional Assessment.

[57] Tessier F., Margaritis I., Richard M.-J., Moynot C., Marconnet P. (1995). Selenium and Training Effects on the Glutathione System and Aerobic Performance. Med. Sci. Sports Exerc..

[58] Huggins R.A., Fortunati A.R., Curtis R.M., Looney D.P., West C.A., Lee E.C., Fragala M.S., Hall M.L., Casa D.J. (2019). Monitoring Blood Biomarkers and Training Load throughout a Collegiate Soccer Season. J. Strength Cond. Res..

[59] Pograjc L., Stibilj V., Falnoga I. (2012). Impact of Intensive Physical Activity on Selenium Status. Biol. Trace Elem. Res..

[60] Rokitzki L., Logemann E., Sagredos A.N., Murphy M., Wetzel-Roth W., Keul J. (1994). Lipid Peroxidation and Antioxidative Vitamins under Extreme Endurance Stress. Acta Physiol. Scand..

[61] Schrauzer G.N. (2000). Selenomethionine: A Review of Its Nutritional Significance, Metabolism and Toxicity. J. Nutr..

[62] Hać E., Krechniak J., Szyszko M. (2002). Selenium Levels in Human Plasma and Hair in Northern Poland. Biol. Trace Elem. Res..

[63] Kim H., Lim H., Lee K., Choi M.H., Kang N.M., Lee C.H., Oh E.J., Park H.K. (2017). Determination of Trace Metal Levels in the General Population of Korea. Int. J. Environ. Res. Public Health.

[64] Jain R., Choi Y. (2015). Normal Reference Ranges for and Variability in the Levels of Blood Manganese and Selenium by Gender, Age, and Race/Ethnicity for General US Population. J. Trace Elem. Med. Biol..

[65] Sesana G., Baj A., Toffoletto F., Sega R., Ghezzi L. (1992). Plasma Selenium Levels of the General Population of an Area in Northern Italy. Sci. Total Environ..

[66] Verlinden M., Van Sprundel M., Van der Auwera J.C., Eylenbosch W.J. (1983). The Selenium Status of Belgian Population Groups: I. Healthy Adults. Biol. Trace Elem. Res..

[67] Smith A., Chang M., Medeiros L. (2000). Generational Differences in Selenium Status of Women. Biol. Trace Elem. Res..

[68] Toro-Román V., Siquier-Coll J., Grijota Pérez F.J., Maynar-Mariño M., Bartolomé-Sánchez I., Robles-Gil M.C. (2024). Plasma, Urinary, Erythrocyte and Platelet Zinc Concentrations in Soccer Players. Nutrients.

[69] Toro-Román V., Grijota F.J., Maynar-Mariño M., Campos A., Martínez-Sánchez A., Robles-Gil M.C. (2024). Plasma, Urinary, Erythrocyte, and Platelet Concentrations of Manganese and Molybdenum in Football Players: Differences between Sexes and during the Season. Appl. Sci..

[70] Stoedter M., Renko K., Hög A., Schomburg L. (2010). Selenium Controls the Sex-Specific Immune Response and Selenoprotein Expression during the Acute-Phase Response in Mice. Biochem. J..

[71] Yoneyama S., Miura K., Itai K., Yoshita K., Nakagawa H., Shimmura T., Okayama A., Sakata K., Saitoh S., Ueshima H. (2008). Dietary Intake and Urinary Excretion of Selenium in the Japanese Adult Population: The INTERMAP Study Japan. Eur. J. Clin. Nutr..

[72] Urbano T., Filippini T., Lasagni D., De Luca T., Sucato S., Polledri E., Bruzziches F., Malavolti M., Baraldi C., Santachiara A. (2021). Associations between Urinary and Dietary Selenium and Blood Metabolic Parameters in a Healthy Northern Italy Population. Antioxidants.

[73] McAdam P.A., Smith D.K., Feldman E.B., Hames C. (1984). Effect of Age, Sex, and Race on Selenium Status of Healthy Residents of Augusta, Georgia. Biol. Trace Elem. Res..

[74] Pinto R.E., Bartley W. (1968). Changes in Glutathione Reductase and Glutathione Peroxidase Activities in Rat Liver Related to Age and Sex. Biochem. J..

[75] Seale L.A., Ogawa-Wong A.N., Berry M.J. (2018). Sexual Dimorphism in Selenium Metabolism and Selenoproteins. Free Radic. Biol. Med..

[76] Schomburg L., Riese C., Renko K., Schweizer U. (2007). Effect of Age on Sexually Dimorphic Selenoprotein Expression in Mice. Biol. Chem..

[77] Letsiou S., Nomikos T., Panagiotakos D.B., Pergantis S.A., Fragopoulou E., Pitsavos C., Stefanadis C., Antonopoulou S. (2014). Gender-specific Distribution of Selenium to Serum Selenoproteins: Associations with Total Selenium Levels, Age, Smoking, Body Mass Index, and Physical Activity. Biofactors.

